# Structural and Functional Characterization of a New Double Variant Haemoglobin (HbG-Philadelphia/Duarte *α*_2_^68Asn→Lys^ *β*_2_^62Ala→Pro^)

**DOI:** 10.5402/2011/735314

**Published:** 2010-11-29

**Authors:** Antonella Fais, Mariano Casu, Paolo Ruggerone, Matteo Ceccarelli, Simona Porcu, Benedetta Era, Roberto Anedda, Maria Carla Sollaino, Renzo Galanello, Marcella Corda

**Affiliations:** ^1^Department of Sciences Applied to Biosystems, University of Cagliari, S.P. Monserrato-Sestu km 0.700, 09042 Monserrato, Italy; ^2^Department of Chemical Sciences, University of Cagliari, S.P. Monserrato-Sestu km 0.700, 09042 Monserrato, Italy; ^3^SLACS, Istituto Officina dei Materiali del CNR and Department of Physics, University of Cagliari, S.P. Monserrato-Sestu km 0.700, 09042 Monserrato, Italy; ^4^Department of Biomedical and Biotecnologies Sciences, University of Cagliari, via Jenner, 09121 Cagliari, Italy

## Abstract

We report the first case of cosegregation of two haemoglobins (Hbs): HbG-Philadelphia [*α*68(E17)Asn → Lys] and HbDuarte [*β*62(E6)Ala → Pro]. The proband is a young patient heterozygous also for *β*°-thalassaemia. We detected exclusively two haemoglobin variants: HbDuarte and HbG-Philadelphia/Duarte. Functional study of the new double variant HbG-Philadelphia/Duarte exhibited an increase in oxygen affinity, with a slight decrease of cooperativity and Bohr effect. This functional behaviour is attributed to *β*62Ala → Pro instead of *α*68Asn → Lys substitution. Indeed, HbG-Philadelphia isolated in our laboratory from blood cells donor carrier for this variant is not affected by any functional modification, whereas purified Hb Duarte showed functional properties very similar to the double variant. NMR and MD simulation studies confirmed that the presence of Pro instead of Ala at the *β*62 position produces displacement of the E helix and modifications of the tertiary structure. The substitution *α*68(E17)Asn → Lys does not cause significant structural and dynamical modifications of the protein. A possible structure-based rational of substitution effects is suggested.

## 1. Introduction

Structural haemoglobin (Hb) variants typically result from a point mutation in a globin gene that produces a single amino acid substitution in the corresponding globin chain. Most of the natural and recombinant haemoglobin variants possess one or two amino acid substitutions on the same polypeptide chain [1] while Hbs with substitutions in both *α* and *β* chains are only occasionally reported [2, 3]. The availability of natural Hb variants offers the opportunity to directly correlate structural and functional modifications to the clinical manifestations [4–6].

Haemoglobin variants are often associated with *α* or *β* thalassaemia, and, while heterozygotes for the variants can manifest limited clinical effects and may be asymptomatic, the double heterozygosity for structural variants and thalassaemia may lead to severe clinical diseases [4–8]. Therefore, a careful analysis of families with interacting haemoglobin variants and *β* thalassaemia is relevant. Moreover, their identification is important for genetic counselling and for structure-function relationship studies. 

Worldwide, more than 800 natural haemoglobin variants have been identified so far [1]. The modifications in their structural properties are responsible for protein instability and may lead to misfolding and precipitation, while alterations in the functional properties are generally associated with altered oxygen affinity, the extent of each phenomenon depending on the location and nature of the substituted amino acid.

During a screening program for the identification of *β*-thalassemia carriers in Sardinia, we identified in a subject heterozygote for the beta zero 39 nonsense mutation a new double variant resulting from tetrameric assembly of the *α* ^68Asn→Lys^ with *β* ^62Ala→Pro^ globin chains. HbDuarte (*α*_2_ *β*_2_^62Ala→Pro^) and HbG-Philadelphia (*α*_2_^68Asn→Lys^ *β*_2_) were previously studied [9–11] but the presence of both substitutions in the same tetramer has not been described before. 

The HbG-Philadelphia/Duarte offers the rare opportunity to characterize a new naturally occurring double Hb variant and to provide some insights into the still open problem of the influence of point mutations on the structure-function relationships in a protein. For example, amino acid substitutions located at *α*68(E17) position such as in HbUbe (*α*_2_^68Asn→  Asp^ *β*_2_) [12, 13] do not influence the functional properties of Hb molecule, while for HbG-Philadelphia two previous papers proposed opposite results [14, 15]: North et al. [14] reported an increased oxygen affinity not detected by Pardoll et al. [15]. HbDuarte has an oxygen affinity higher than the HbA and a moderate instability [9, 11]. 

Additionally, we have the chance to assign the true contribution of two specific amino acid substitutions on determining the functional and structural behaviour of the double variant compared with the single variants Hbs once these latter have been well characterized. In other words, we might discriminate whether the double substitutions affect additively the properties of the system, that is, the final effect is the sum of the single effects, or additional intereffects come into play subtly producing new features. Finally, it would be useful to extract some structural-dynamical criteria to foresee the perturbations introduced in the systems by multiple mutations. 

Using a series of approaches, including molecular biology and protein studies, we carried out the investigation on the purified double variant haemoglobin. In addition, proton Nuclear Magnetic Resonance (NMR) spectroscopy and Molecular Dynamic (MD) simulations assessed the effects of the amino acid substitutions on the tertiary and quaternary structure, as well as on the dynamical fluctuations.

## 2. Materials and Methods

### 2.1. Blood Samples

Venous samples from the proband and from his parents were collected using heparin or EDTA as an anticoagulant. Cells were washed three times with an iso-osmotic NaCl solution by centrifugation at 1000 g, and the packed cells were lysed by adding distilled water in a 1 : 1 ratio. After incubation at 4°C, 1 vol. of CCl_4_ was added, and the solution was then centrifuged for 30 min at 12000 g to remove the ghosts.

### 2.2. DNA Sequences of Globin Genes

DNA was extracted from EDTA peripheral blood samples with saline method. To define the mutations, DNA of *α* and *β* globin genes was amplified by PCR [16] and directly sequenced using ABI PRISM DNA 3100 (Applied Biosystem).

### 2.3. Haemoglobin Analysis

The red cells lysate was analysed by IEF in 5% thin layer polyacrylamide gels (Pharmalyte pH range 6.7–7.7 Amersham Pharmacia Biotec AB) [17]. The abnormal haemoglobins were identified and quantified by HPLC (Variant I, Bio-Rad, Milan, Italy). Dissociated globin chains were analysed in polyacrylamide gels in the presence of 5% acetic acid, 8M urea and Triton X-100 (AUT-PAGE) [18–20] and by RP-HPLC (Agilent 1100 series) on a Zorbax 300SB-C18 column (Agilent Technologies). Each chromatogram was developed at room temperature with a linear gradient from 60% to 80% of the solvent A (50% acetonitrile, 20% methanol, 30% NaCl 155 mM) in solvent B (25% acetonitrile, 40% methanol, 30% NaCl 155 mM). The solvent program was a 90 min gradient with a flow rate of 1.4 mL/min. Absorbance was monitored at 215 nm. HbDuarte, double variant HbG-Philadelphia/Duarte present in the proband lysate, and HbG-Philadelphia from blood cells donor carrier for this variant were purified by ion-exchange chromatography (IEC) using a HiLoad Q column (Amersham Pharmacia Biotec AB). The column was first equilibrated with 20 mM Tris-HCl buffer, pH 8.0; then, the pH was decreased to 7.0 with a linear gradient. Absorbance was monitored at 280 nm. The purity of variant Hbs was checked by IEF and RP-HPLC. The separated fractions were analysed by means of RP-HPLC in order to highlight the presence of possible hybrids *αα* ^68Asn→Lys^ *β*_2_^62Ala→Pro^. 

### 2.4. Functional Studies

Oxygen equilibrium curves were obtained at 25 and 37°C, in the pH range 7.0–8.0, at Hb concentration of ~80 *μ*M on a haem basis by tonometric method [21]. An average standard deviation (SD) of ±3% for values of p_50_ was evaluated. The p_50 _is the partial pressure of the ligand at which 50% of the haems are oxygenated.

Organic phosphates were removed from Hb using a Sephadex G-25 column equilibrated with 100 mM Tris/HCl buffer pH 8.0 containing 100 mM NaCl.

Functional experiments were carried out in 100 mM Tris or BisTris/HCl buffers, containing 100 mM NaCl, in the presence and in the absence of 5 mM 2,3-diphosphoglycerate (2,3DPG). Hill graphics [22] were built by plotting each individual log  (*y*/1 − *y*) versus the corresponding log pO_2_, where *y*/1 − *y* is the “fractional” saturation of Hb in oxygen, that is, the ratio between Hb saturated and nonsaturated in O_2_. The oxygen affinity in terms of p_50_ and cooperativity of oxygen binding as indicated by the value of the Hill coefficient *n*_50_ values were calculated by linear regression from Hill equation for oxygen saturation levels between 40% and 60%. The magnitude of the heterotrophic effects was calculated as a Δp_50_ ± the effector.

The methaemoglobin content was calculated from optical spectrum recorded at the end of oxygen equilibrium measurements.

### 2.5. ^1^H NMR Spectroscopy Investigation

The ^1^H NMR spectra were recorded on a Varian Unity-Inova spectrometer at a resonance frequency of 399.948 MHz. All Hb samples (~3%) were dissolved in 100 mM sodium phosphate buffer (10% D_2_O) at pH 7.0. The experiments were performed at 29.0 ± 0.1°C for Hb in the CO form and in the deoxy form. All the ^1^H NMR experiments were carried out on a 5 mm Wilmad high-pressure NMR tubes (OD 5 mm and ID 4.2 mm) using 3.7 *μ*s pulse (90°), 1 s repetition time, and spectral width of 12 kHz for CO form and 80 KHz for deoxy form. Suppression of the intense water signal was achieved by direct saturation during the relaxation delay. The accuracies of chemical shift measurements in our samples, determined by repeating the experiments with three different samples, are ±0.08 ppm for the resonances in the CO form and in the range 10–25 ppm for the resonances in the deoxy form. Chemical shifts in all spectra were referenced to DSS (2,2-dimethyl-2-silapentane-5-sulfonate) through the water signal set at 4.80 ± 0.05 ppm.

### 2.6. Protein Structural Analyses

We used state-of-the-art MD simulations at an all-atom level to investigate structural rearrangements of the protein at the atomic scale. We started with the T deoxy form (PDB code: 1 hbhb; resolution 1.74 Å [23]) solvated in a truncated octahedron cell with initial size of 80 Å, resulting in a system with 9500 water molecules (~37000 atoms). The simulation scheme was successfully used also in the study of myoglobin [24–26]. We refer to [11] for all details on the simulation protocol. At the end of the relaxation cycle (3 ns), we introduced by hand the mutations. We prepared two independent systems: (i) the double variant substituting the alanine amino acid in proline at the positions E6 of the *β* chains and the asparagine to lysine at position E17 of the *α* chains, (ii) the Philadelphia variant substituting only the asparagines. HbA and mutated systems were simulated for additional 6 ns (HbA), 8 ns (double variant), and 1.5 ns (HbG-Philadelphia), saving data every 100 fs. This procedure allows us to follow relaxation of both systems and to compare them to possibly point out the structural and dynamical perturbations induced by the mutation.

## 3. Results

### 3.1. DNA and Hb Analyses

The hemolysate of proband checked by HPLC revealed the features of two main Hbs species, the first one (~46.2%) corresponding to Hb Duarte (that has a mobility like HbA) and the second (~39.9%) to Hb G-Philadelphia.SLACS-Istituto Officina dei Materiali del CNR and Department of HbA_2_ and HbF accounted for 3.8% and 1.30% of the total amount, respectively (data not shown). 

**Figure 1:**
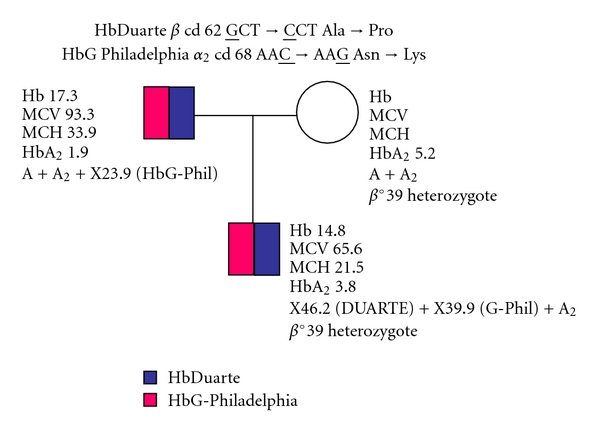
Family tree and haematological data.

**Figure 2:**
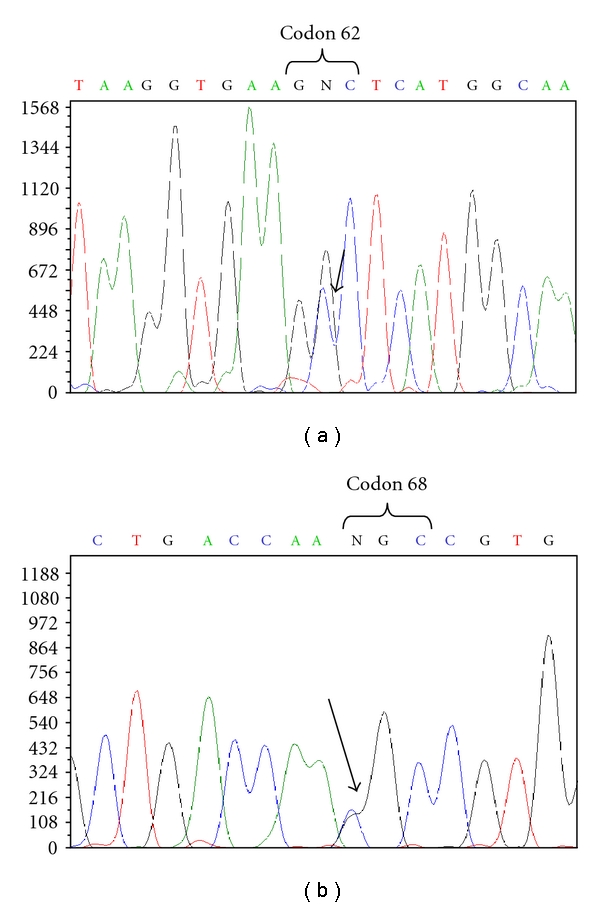
Sequence analysis (ABI PRISM 3100- Applied Biosystem) of globin genes. (a) *β*-globin gene coding strand of the proband showing that he was heterozygous for Hb Duarte GCT→CCT (*β*62Ala → Pro). (b) *α*-globin gene coding strand of the proband showing that he was heterozygous for Hb G-Philadelphia AAC→AAG (*α*68Asn → Lys).

The family tree and the haematological data are described in Figure 1. The proband analysis of amplified DNA pointed out the presence of a nonsense mutation at codon 39 in the heterozygous state (data not shown). Direct DNA sequencing of amplified *β* globin genes revealed a mutation at codon 62 GCT→CCT (Figure 2(a)) compatible with an Ala → Pro substitution, while sequencing *α* globin genes unveiled a single nucleotide substitution: AAC→AAG at codon 68 of *α*_2_ gene (Figure 2(b)), which results in a Asn → Lys substitution. All these experimental data, in agreement with the presence of the heterozygosity for *β* ^0^-thalassaemia, suggested that the hemolysate is composed by two tetramer molecules, namely, *α*_2_ *β*_2_^62Ala→Pro^ (HbDuarte) and *α*_2_^68Asn→Lys^ *β*_2_^62Ala→Pro^, the new double variant named HbG-Philadelphia/Duarte.

**Figure 3:**
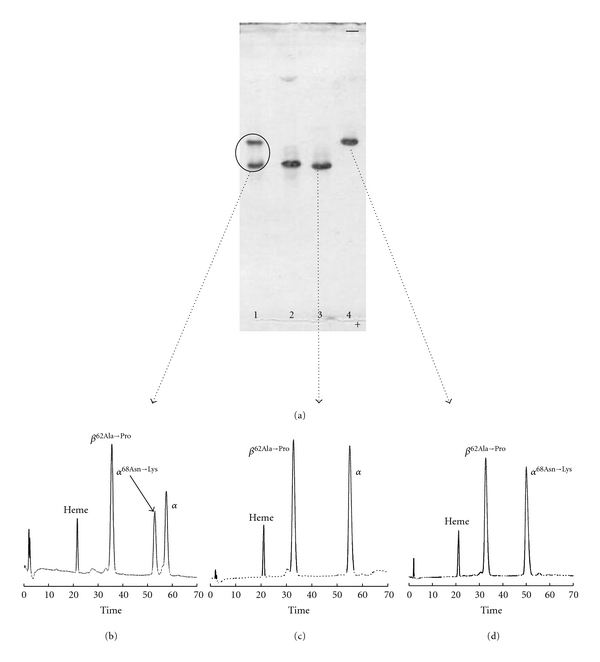
Identification of variant hemoglobins. (a) IEF of hemolysate variant (lane 1), HbA (lane 2), purified Hb Duarte (lane 3), and purified Hb G-Philadelphia/Duarte (lane 4); RP-HPLC of the globin chains of (b) hemolysate variant; (c) purified Hb Duarte; (d) purified Hb G-Philadelphia/Duarte.

IEF procedure confirmed the presence of two haemoglobin components (Figure 3(a) lane 1), one of which had an electrophoretic mobility similar to that of HbA (Figure 3(a) lane 2), the other migrating more slowly. RP-HPLC analysis, as shown in Figure 3(b), exhibited an arrangement of the hemolysate in three globin chains, two *α* like and one *β* like [27]. 

To further verify this hypothesis the haemoglobin components were purified by IEC and analysed by IEF (Figure 3(a) lanes 3 and 4). The Hb component that moved like HbA (Figure 3(a) lane 3) was identified as HbDuarte [9], the other component as the double variant (*α*_2_^68Asn→Lys^ *β*_2_^62Ala→Pro^) (Figure 3(a) lane 4). Globin chains analysis by RP-HPLC of the two haemoglobins purified supported the electrophoretic results (Figures 3(c) and 3(d)) confirming that the two haemoglobin components of the hemolysate were purified and identified as *α*_2_ *β*_2_^62Ala→Pro^ (HbDuarte) (Figure 3(c)) and as a double variant Hb, consisting of *α*_2_^68Asn→Lys^ *β*_2_^62Ala→Pro^ (Figure 3(d)). From the analysis of the purified haemoglobins the presence of any hybrid species (*αα*_2_^68Asn→Lys^ *β*_2_^62Ala→Pro^was ruled out, as it can be seen in Figures 3(a) and 3(b). 

The presence of haemoglobin with both *α* and *β* mutations did not produce apparently any particular clinical effect, being the patient asymptomatic. The proband presented thalassaemia-like hematological features, reduced mean corpuscular volume (MCV 65.6 fl), mean corpuscular haemoglobin (MCH 21.5 pg), increased HbA_2 _(3.8%), reticulocytes 2.6 (×1000), but haemoglobin concentration (14,8 g/dl) was higher as compared to beta thalassemia carriers.

### 3.2. Functional Studies

The oxygen affinity in terms of log p_50_ and cooperativity (*n*_50_) of purified HbG-Philadelphia/Duarte are reported in Table 1 in comparison with HbA. The ratio of p_50_(HbA)/p_50_(HbG-Philadelphia/Duarte) ranged from 2.63 to 1.77 depending on pH and organic phosphate, this being indicative of the higher O_2 _affinity of the double variant Hb. The cooperativity of oxygen binding value and the alkaline Bohr effect (Δlog p_50_/ΔpH) measured in absence and in the presence of 2,3-DPG slightly diminished with respect to that of native human HbA.

**Figure 4:**
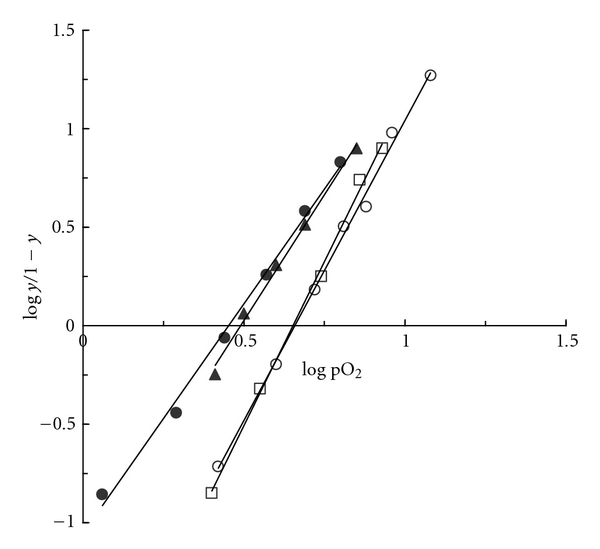
Hill plots of purified Hbs. (●) Hb G-Philadelphia/Duarte, (▲) Hb Duarte, (□) Hb G-Philadelphia and (O) HbA. Experimental conditions: 0.1 M BisTris/Tris 0.1 + 0.1 M NaCl buffer at pH 7.4, 25°C. O_2 _pressure is expressed in Torr units.

Figure 4 reveals a shift of the Hill plot of HbG-Philadelphia/, and HbDuarte purified toward smaller values with respect to HbG-Philadephia and HbA. The data indicate that purified HbG-Philadelphia/Duarte and HbDuarte are characterized by undistinguishable O_2_ binding properties but different from those of purified HbG-Philadelphia and HbA.

The amount of met-Hb contained in the samples, after oxygen binding experiments, ranged from 2% to 7% of total Hb.

### 3.3. NMR Studies

^1^H NMR spectra of purified Hb G-Philadelphia (*α*_2_^68Asn→Lys^ *β*_2_) and Hb G-Philadelphia/Duarte (*α*_2_^68Asn→Lys^ *β*_2_^62Ala→Pro^) in the deoxy (T) and CO (R) form were compared to those of HbA to define the effects of amino acid substitutions on the heme environment and on subunit interface interactions.

**Figure 5:**
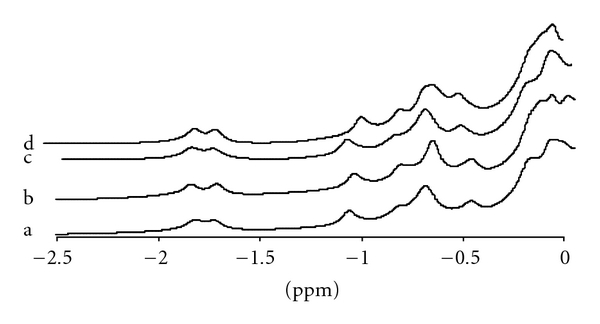
Hyperfine shifted and exchangeable proton resonances of Hbs purified in the CO form: (a) HbA, (b) Hb Duarte, (c) Hb G-Philadelphia, and (d) Hb G-Philadelphia/Durate.

The substitution *α*68Asn → Lys does not modify the NMR spectra of both HbG-Philadelphia and HbG-Philadelphia/Duarte in the CO form (R state), as demonstrated in Figure 5. Instead, for the deoxy form the same amino acid substitution is responsible for remarkable modifications in the spectra, as discussed in the following. 

**Figure 6:**
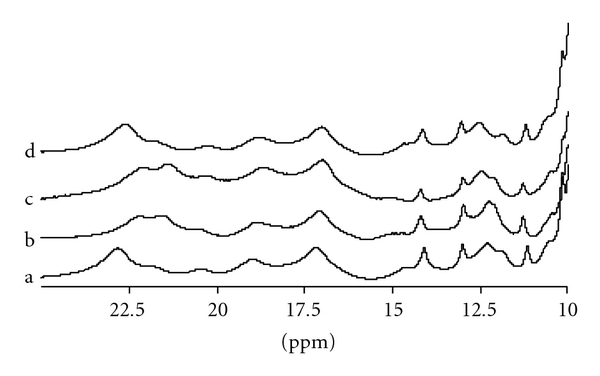
Hyperfine shifted and exchangeable proton resonances of Hbs purified in the deoxy form. (a) HbA, (b) Hb Duarte, (c) Hb G-Philadelphia/Duarte, and (d) Hb G-Philadelphia.

The ^1^H-NMR spectra resonances of HbA, HbG-Philadelphia/Duarte and HbG-Philadelphia in the deoxy form from 10 to 25 ppm together with the recently published [11] and unveiled ^1^H-NMR spectrum of HbDuarte (*α*_2_ *β*_2_^62Ala→Pro^) are reported in Figure 6 to better identify changes and similarities. The resonances in the ranges 14.5–25 ppm and 11.5–13.5 ppm originated from protons subjected to significant hyperfine interactions with the unpaired electrons of the Fe atom, that is, from protons belonging to the heme groups and/or to residues located in the heme pocket.

As shown in Figure 6, some changes were observed in the HbG-Philadelphia spectrum in the range 11.5–13.5 ppm compared to HbA: in particular, the signal at ~12.2 ppm in HbA is shifted to ~12.6 ppm in HbG-Philadelphia. The hyperfine shifted resonances at ~12.2 ppm, 14.6 ppm, 21.8, and 22.8 ppm of the HbG-Philadelphia/Duarte spectrum exhibited modifications of the chemical shift with respect to HbA. The last two resonances observed also in the ^1^H NMR spectrum of the HbDuarte [11] were assigned to protons in the *β* chains of deoxy HbA [28].

Clearly, most of the changes observed in the spectrum of the double variant can be ascribed to the substitution *β*62Ala → Pro. This substitution causes an adjustment in the configuration of the amino acid residues (and/or of the heme groups) in the *β* chains, which sense the hyperfine interactions with the heme iron [11]. In the range 16–21 ppm, no changes were observed, the resonances at ~18.7 ppm and at ~17.1 ppm being associated with protons in the *β* chains and in the *α*-chains of deoxy HbA, respectively [28]. Only the shift of the signal at ~12.2 ppm in HbG-Philadelphia/Duarte was similar to that observed in HbG-Philadelphia, suggesting that structural changes in the *α* chains originate this perturbation. 

Several exchangeable proton features in the range 10–15 ppm were recognizable in the ^1^H NMR spectrum of deoxy HbA. The resonances at 14.1 ppm and 11.2 ppm were identified as due to the intersubunit H-bond between *α*42Tyr and *β*99Asp and between *α*94Asp and *β*37Trp in the *α*_1_ *β*c_2_ interface. The analysis of these proton resonances gave indication of the quaternary conformational modifications in the T state, as a consequence of residues substitutions [29–33]. In the deoxy state of HbA the H-bond between *α*103His → *β*131Gln at the interfaces between *α*_1_ *β*_1_ subunits generated the resonance at 13.2 ppm. All these resonances in the HbG-Philadelphia spectrum coincided with those observed in the HbA spectrum, indicating that an eventual link between *α*_2_^68Asn→Lys^ *β*_2_ substitution and perturbations at the level of the *α*_1_ *β*_1_ and *α*_1_ *β*_2_ subunits interfaces can be certainly ruled out. The resonances at 14.1 ppm and 11.2 ppm in HbG-Philadelphia/Duarte were slightly shifted toward low fields, as already observed in the HbDuarte [11]. In agreement with what previously discussed for HbDuarte, this observation can be attributed to minor adjustments of the *α*_1_ *β*_2_ interface [11] influenced by the *β*62Ala → Pro mutation.

### 3.4. Structural Analyses

The substitution *β*62Ala → Pro in HbDuarte induced structural modifications as already reported in a previous paper [11], whereas the substitution *α*68Asn → Lys in HbG-Philadelphia does not seem to have a significant influence on the structure. In the wild type (WT), the residue *α*68Asn forms a hydrogen bond with *α*79Ala at position EF8, in particular with the carbonyl backbone group. The latter does not form an intrahelical hydrogen bond in the X-ray structure, Ala79 being a defect point of the helix F. Accordingly, this hydrogen bond is not constant all over our simulations, because it is detectable for only 50% of the simulation, and the interaction is not critical for both helix E and F. After substitution, being Lys a charged residue and with a longer tail, two possible hydrogen bonds can be formed either with Ala79 (30%) or with Asp64 (70%), at position E13 on the helix E. The lateral chain of Asp64 in WT points toward the solvent, and no crucial interaction with other amino acids is established.

**Figure 7:**
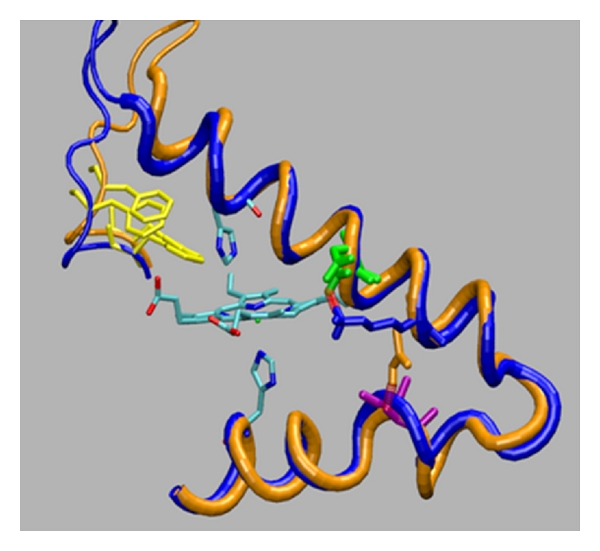
Pictorial view of the alpha globin chains superimposed after 6 and 8 ns of MD simulations. (Blue tube) double variant and (orange tube) HbA. In yellow the amino acids of the CD corner pointing the distal histidine. In green the Glu64 at position E13, in violet Ala79 at position EF8, and in orange and blue the residue at position E17, respectively, Lys and Asn, for the two systems.

As we can see from Figure 7, where we superimpose a WT structure (after 6 ns) with the HbG-Philadelphia/Duarte structure (after 8 ns), there are minimal changes in the region of *α*68. In particular, the distal region, which in the HbDuarte showed important deviations, is unaffected by the G-Philadelphia mutation (yellow amino acids in Figure 7).

**Figure 8:**
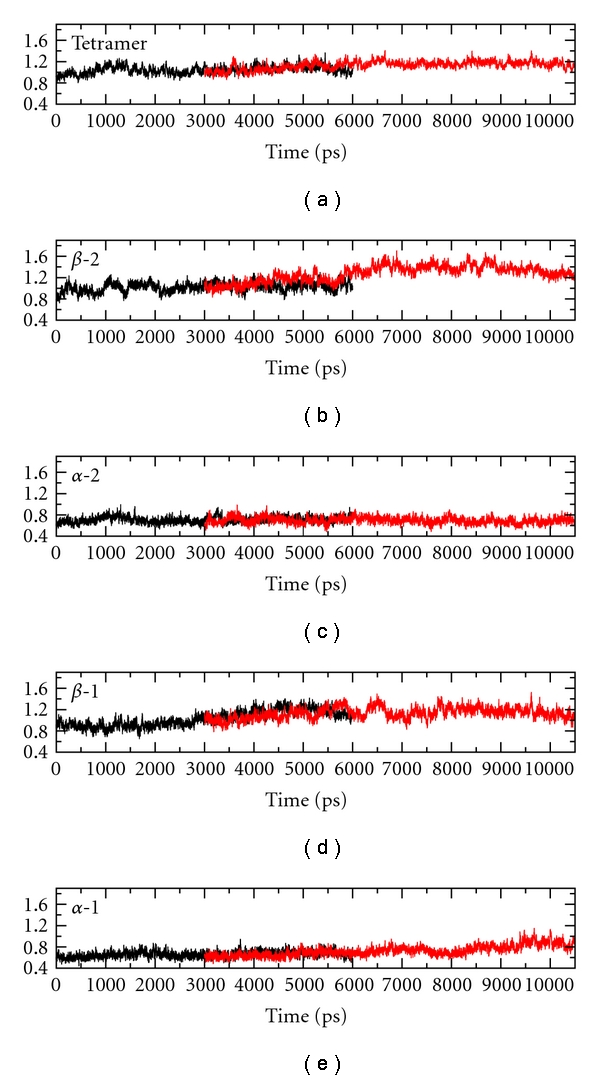
Xrms of (black curves) Deoxy HbA and (red curves) HbG-Philadelphia/Duarte. (a) Tetramer, (b)–(e) *β*2, *α*2, *β*1, and *α*1 chains, respectively.

Inspection of the deviations of our mutated structure from X-ray, when compared with the WT simulation, does not show any relevant deviations on the alpha subunits and on the whole tetramer (see Figure 8). The results obtained with short (1.5 ns) simulations of HbG-Philadelphia agree with the above analysis. The substitution Asn → Lys at the position E17 does not create any local rearrangements near the heme group nor at the *αβ* interfaces.

## 4. Discussion

A structural analysis of the two variants in the proband was carried out at both protein and DNA levels, demonstrating the presence of a new haemoglobin double variant never described before, which we named HbG-Philadelphia/Duarte or *α*_2_^68Asn→Lys^ *β*_2_^62Ala→Pro^. The study of the proband's family pointed out that the mother was a carrier of *β* ^0^-thalassemia and the father of HbDuarte and HbG-Philadelphia. Only few natural haemoglobin variants carrying two substitutions in the same globin chain have been reported, and the probability to find a double mutation in the two different polypeptide chains is extremely low [2, 3]. 

The simultaneous occurrence of such a double variant in *α* and *β*  chains offers the excellent opportunity to investigate to which extent the effects of the double mutations sum up, once the perturbations associated with each single variant are well characterized. This could also be intended as an attempt to search structural boundaries that fix the range of influence of given mutations on tertiary and quaternary structures. Such a study should help to map regions central to specific functionalities. Note that both substitutions considered in our study are localized in the E trait, which sandwiches with the F helix the heme prosthetic group. Hence, displacements in this region could weaken the H-bonds connecting the E to the A, F, and H helices destabilizing the tertiary folding of globin chains. This might lead to a rearrangement of the heme pocket near helix E where oxygen binds, determining variations of functional properties. In particular, the *α*68(E17)Asn is involved in two intrachain contacts with *α*79(EF8)Ala and *α*80(F1)Leu. These contacts serve to anchor the EF clamshell holding the heme. Additionally, the E helix is connected to A helix via a linkage between *α*67(E16)Thr and *α*14(A12)Trp, and the F helix to H helix via a linkage between the backbone carbonyl group of *α*93(FG5)Val and *α*140(HC2)Tyr. The residue *β*62(E6), instead, is close to the distal heme-linked histidine (E7), and it is involved in maintaining the E helix. An important question is how and to which extent these two substitutions contribute to influence the structural and/or functional properties in the Hb tetramer.

The whole corpus of our results indicates clearly that the structural modifications responsible for the functional behaviour of the new double variant Hb must be ascribed exclusively to the *β*62Ala → Pro ruling out a possible involvement of the *α*68Asn → Lys mutation. The oxygen affinity of HbG-Philadelphia/Duarte increases; its cooperativity and the Bohr effect slightly decrease. These functional abnormalities are attributed to the single *β* mutation, the same characterizing the HbDuarte, since the second substitution in *α* chain appears unable to determine functional alteration. In fact, in HbG-Philadelphia the substitution of the polar uncharged Asn residue in *α*68 with the positively charged and longer Lys does not alter the functional properties with respect to those of HbA (Figure 4). In the Hb Duarte the substitution of the alanine with a proline residue produces an increase in the oxygen affinity similar to the one observed in HbG-Philadelphia/Duarte [11]. These functional results are supported by extensive structural investigations via NMR and computer simulations. According to our NMR data, the T state and the R state intersubunit contacts are unaffected by the *α*68Asn → Lys mutation in the HbG-Philadelphia/Duarte and HbG-Philadelphia. This suggests that the features of the quaternary transition *R*  ↔  *T* are preserved upon mutation as confirmed by the almost unaltered cooperativity, DPG and Bohr effect detected in functional experiments. On the contrary, ^1^H NMR data on the HbG-Philadelphia/Duarte shows differences in the tertiary structure around the heme pockets. In fact, the chemical shift at ~12.2 ppm attributed to the *α* chains changes upon 68Asn → Lys mutation similarly to what observed in the HbG-Philadelphia. The other shifts observed at ~14.6 ppm, ~22.8, and ~21.8 ppm in Hb G-Philadelphia/Duarte are at the same position than those observed for HbDuarte. The abnormal functional properties of HbG-Philadelphia/Duarte can be assigned to the *β*62Ala → Pro rather than to the other substitution *α*68Asn → Lys present in HbG-Philadelphia, which has been well demonstrated to be functionally latent (see Figure 4). In this latter haemoglobin, as in HbUbe 2 [12, 34], the site of amino acid substitution is considerably far from the distal histidine of the *α* chain (His *α*58), which is directly related to the function of heme iron.

A further support to the NMR analysis comes from the MD simulations that encompass microscopic features barely seen in experiments. According to MD results on HbG-Philadelphia/Duarte and HbG-Philadelphia, the pattern of intrachain contacts in the *α* chain is conserved and remains stable when Asn is substituted by Lys (Figure 7).

In tune with the search of structural thresholds identifying the effects of mutation on specific functions, the literature offers some hints: amino acid substitutions in the E trait of *α* chain do not affect significantly the functional properties [1]. Another mutant at the same position of HbG-Philadelphia, HbUbe 2 (*α*_2_^68Asn→Asp^ *β*_2_) [12, 34], presents unaltered affinity despite the change of charge carried by residue at E17*α*. It seems very difficult to modify functionalities by acting on the trait E of the intrahelix contacts, which maintain tightly the *α* chain tertiary structure. 

The situation is profoundly different for the *β* chains: *β* substitutions in E trait only occasionally do not affect a functional behaviour [1]. In addition, since proline can only be tolerated as one of the first three residues of a regular helix, its introduction in the middle of a helical segment usually leads to a destabilization of the subunit. In most cases, this results in a modification of functional properties and/or stability of the tetramer as indicated by several investigations on natural Hb variants [1, 35]. This is the situation characterizing the double variant investigated here. The proline at the *β*62 (E6) position, close to the distal haem-linked histidine (E7), produces a destabilization of the E helix toward the E1 residue that extends downward to the CD corner. The outcome is an alteration of the haem cavity structure remarkably mirrored in the functional properties of the protein molecule.

## 5. Conclusions

In conclusion, the results on the double variants and those available for the single mutations permit to point out some aspects: (1) both mutations take place in the E trait; the *β* one influences structural and functional properties that in the *α* chain are practically silent; (2) the *α* and *β* substitutions in HbG-Philadelphia-Duarte act independently; (3) along the E trait the functional perturbation is strictly related to the location of the substitution: the 68Asn → Asp mutation in *α* chain do not give apparent changes; the 62Ala → Pro in the *β* chain give remarkable effects. 

Our data and the information available to our knowledge in the literature suggest that the relative position on the globin chains (at least in the E trait), rather than the kind of amino acid substitution, plays a more important role in determining structural and functional characteristics of the haemoglobin molecule. However, the identification of a set of residues whose substitution, single or combined, causes functional and structural changes in different degree, could be useful for engineering an Hb variant suitable as an artificial oxygen carrying fluid.

## Figures and Tables

**Table 1:** Oxygen binding properties of purified HbG-Philadelphia/Duarte.

	pH	log p_50_	*n*_50_	p_50_HbA/p_50_HbG Philadelphia/Duarte
	6.47	0.81 (1.13)	2.56 (2.71)	2.09
−5 mM 2,3-DPG	7.05	0.67 (0.94)	2.54 (2.67)	1.86
	7.4	0.41 (0.66)	2.64 (3.0)	1.77
Alkaline Bohr effect		−0.43 (−0.50)		

	6.54	1.09 (1.47)	2.40 (2.74)	1.40
+5 mM 2,3-DPG	7.18	0.88 (1.30)	2.29 (2.78)	2.63
	7.44	0.67 (1.0)	2.45 (2.96)	2.14
Alkaline Bohr effect		−0.46 (−0.52)		

Corresponding values for HbA are given in parentheses.

Data were obtained with 0.1 M Bis-Tris/Tris buffer + 0.1 M NaCl at 25°C. O_2_ pressure is expressed in Torr units.

An average S.D. of 5% for values of log p_50_ was calculed.

## Abbreviations

Hb: Haemoglobin
HbA: Human adult haemoglobin
NMR: Nuclear Magnetic Resonance
MD: Molecular dynamics
WT: Wild type
Xrms: Instantaneous mean-square deviation of C_*α*_ carbons from their X-ray structure
IEF: Isoelectric focusing.
